# Sleep Quality Assessment in Intensive Care Units: Comparing Actigraphy and the Richards Campbell Sleep Questionnaire—A Pilot Study in the Moroccan Context

**DOI:** 10.3390/clockssleep7030049

**Published:** 2025-09-16

**Authors:** Abdelmajid Lkoul, Keltouma Oumbarek, Youssef Bouchriti, Asmaa Jniene, Tarek Dendane

**Affiliations:** 1 Laboratory of Biostatistics, Clinical Research and Epidemiology, Faculty of Medicine and Pharmacy, Mohammed V University in Rabat, Rabat 10010, Morocco; oumbarekeltouma@gmail.com (K.O.); dendanetarek@gmail.com (T.D.); 2 Geosciences, Environment and Geomatic Laboratory, Department of Earth Sciences, Faculty of Sciences, Ibn Zohr University, Agadir 80000, Morocco; 3 Laboratory of Physiology, Exercise Physiology and Autonomic Nervous System Team, Faculty of Medicine and Pharmacy, Mohammed V University in Rabat, Rabat 10010, Morocco; a.jniene@um5r.ac.ma; 4 Department of Pulmonology, Ibn Sina Hospital Center, Mohammed V University in Rabat, Rabat 10010, Morocco

**Keywords:** sleep, actigraphy, Richards–Campbell sleep questionnaire, intensive care unit

## Abstract

Sleep in intensive care unit (ICU) patients is frequently disrupted, which may adversely affect their overall health and recovery. Despite the implementation of various strategies to promote sleep, accurately assessing its quality remains complex. This pilot study aimed to evaluate both the quality and quantity of sleep in ICU patients using actigraphy (ACT) and the Richards–Campbell Sleep Questionnaire (RCSQ) and to compare the diagnostic performance of these two tools. We conducted a prospective observational study including 228 ICU patients. Sleep was assessed using both RCSQ and ACT. Receiver Operating Characteristic (ROC) curve analysis was used to evaluate the discriminative ability of each tool (Area Under the Curve [AUC], sensitivity, specificity), with optimal cut-off points determined using Youden’s Index. The Mann–Whitney U test was used to compare sleep parameters between patients classified as having good or poor sleep based on ACT measurements. The mean RCSQ score was 38.16 ± 17.09, indicating poor perceived sleep quality. Sleep onset latency (based on RCSQ) was 35.71 ± 21.44 min, with a mean of 40.32 ± 20.03 awakenings. According to ACT, sleep latency was 39.23 ± 22.09 min, and total sleep duration was 198.15 ± 128.42 min (approximately 3 h and 18 min), which is significantly below recommended levels. The average number of awakenings recorded was 24.85. In terms of diagnostic performance, the RCSQ demonstrated excellent discriminative ability (AUC = 1.00 for the total score), while ACT showed more variable results: total sleep duration had a good AUC of 0.91, while sleep latency showed a lower performance with an AUC of 0.50. The RCSQ proved to be more reliable than ACT in assessing sleep quality in ICU patients, providing consistent results across multiple parameters, including sleep depth, latency, and number of awakenings. Conversely, ACT yielded less consistent findings, particularly regarding sleep latency and nighttime interruptions. Further studies are warranted to refine objective tools for evaluating sleep in critically ill patients.

## 1. Introduction

Sleep disorders are common among patients in intensive care units (ICUs), significantly affecting their health and overall well-being [1]. Numerous studies have highlighted profound alterations in sleep architecture in this population, such as increased sleep fragmentation, frequent awakenings, and lighter sleep stages [2,3]. These sleep abnormalities are not only frequent but have also been associated with higher mortality rates and poorer clinical outcomes among hospitalized patients [4]. Furthermore, research has revealed disruptions in circadian rhythms, prolonged daytime sleep, and a high incidence of ICU delirium, all of which contribute to the neuropsychological burden experienced by these patients [5,6]. Patients often perceive sleep disturbances as a significant source of stress, and their effects may persist long after ICU discharge, contributing to post-intensive care syndrome symptoms, including cognitive and psychological impairments in survivors [6,7].

Polysomnography (PSG) is currently considered the gold standard for evaluating sleep quality in the ICU environment [8,9]. However, this method presents several limitations, including its technical complexity, high cost, and time-consuming procedures. In addition, the presence of abnormal electroencephalographic (EEG) activity in critically ill patients raises questions about the applicability and reliability of the standard scoring system recommended by the American Academy of Sleep Medicine in this specific context. These limitations have led to the exploration of alternative methods to assess sleep in ICU patients [9].

In hospitals, actigraphy has been used to estimate sleep quantity and circadian rest–activity rhythms when PSG is impractical at scale, with mixed accuracy depending on illness severity and nighttime care interruptions [10,11]. In the ICU, measurement is further complicated by sedation, mechanical ventilation, neuromuscular blockade, delirium, and prolonged immobility, which may reduce the information content of movement signals and limit the feasibility of PSG [12]. Actigraphy (ACT), a widely recognised non-invasive technique, has emerged as a promising alternative to PSG. This method records motor activity during sleep, allowing for the measurement of body movements at predefined intervals (e.g., 15, 30, or 60 s). The data collected via accelerometry is stored and analysed using specific algorithms. The choice of algorithm, adapted to the specific patient population, is crucial to ensure the sensitivity and specificity of the assessment. Despite its ease of deployment and cost-effectiveness, ACT has limited validity for sleep–wake discrimination in critically ill adults when assessed against the reference standard of polysomnography and tends to misclassify motionless wakefulness as sleep [10]. In clinical practice, ACT is often used in combination with other methods, particularly to evaluate the impact of clinical interventions on sleep.

In addition to objective measures, subjective assessment of sleep quality is commonly performed using questionnaires. The Richards–Campbell Sleep Questionnaire (RCSQ) is among the most widely used instruments in this context. It consists of five items assessing different dimensions of sleep, namely sleep depth, sleep latency, number of awakenings, ease of returning to sleep, and overall sleep quality, along with an optional item evaluating noise disturbances [13].

This study aimed to assess both the quality and quantity of sleep in ICU patients using both the Moroccan Arabic version of the RCSQ (AM-RCSQ) and ACT and analyze the diagnostic accuracy of these two assessment tools.

## 2. Results

Descriptive results of the sample are presented in Table 1. The mean age of patients was 54.25 ± 16.76 years, indicating notable variability. The mean body mass index (BMI) was 23.13 ± 4.86 kg/m^2^, suggesting a generally normal weight profile. The pain score (VAS) averaged 4.07 ± 3.24, indicating moderate pain intensity among most patients, with considerable inter-individual variability. The Charlson Comorbidity Index had a mean of 1.25 ± 1.37, while Quick SOFA and APACHE II scores averaged 0.73 ± 0.85 and 10.66 ± 5.76, respectively.

The average ICU length of stay was 5.44 ± 2.53 days, with significant variation among patients. AM-RCSQ scores revealed a generally poor perception of sleep quality:

Perceived sleep depth: 39.03 ± 22.43, sleep latency: 35.71 ± 21.44, number of awakenings: 40.32 ± 20.03, return to sleep: 38.88 ± 18.81, overall sleep quality: 38.93 ± 17.12, Noise/disturbance: 33.23 ± 17.72. The overall AM-RCSQ score was 38.16 ± 17.09, reflecting a generally unsatisfactory quality of sleep in this ICU population.

In contrast, actigraphy data showed slightly different trends. Sleep latency (ACT): 39.23 ± 22.09 min, total sleep duration: 198.15 ± 128.42 min (approximately 3.3 h), which is well below the recommended levels for restorative sleep. Sleep interruptions: 52.28 ± 24.85 episodes per night, and overall sleep efficiency score: 37.03 ± 19.2. These actigraphic findings corroborate the AM-RCSQ results, confirming poor sleep quality in this critically ill population.

Table 2 provides a detailed analysis of the comparative diagnostic performance of the AM-RCSQ and actigraphic parameters. Specifically, it assesses their ability to discriminate between patients with good sleep (*n* = 64) and those with poor sleep (*n* = 164). To this end, several key indicators were examined: the optimal cut-off values determined using Youden’s Index, the area under the ROC curve (AUC) with its standard deviation (SD), *p*-value, as well as the sensitivity, specificity, and diagnostic accuracy percentages.

Firstly, regarding the AM-RCSQ, the results demonstrate that sleep depth (threshold = 53) shows robust diagnostic performance (AUC = 0.84; SD = 0.0204; *p* < 0.001). This parameter combines a sensitivity of 82% with a specificity of 85%. Similarly, sleep latency (threshold = 43) exhibits comparable performance (AUC = 0.86; SD = 0.0130; *p* < 0.001), although its profile favours higher sensitivity (92%) at the expense of slightly lower specificity (80%). Furthermore, the number of awakenings (threshold = 57) exhibits excellent discriminative power (AUC = 0.85; SD = 0.0257; *p* < 0.001), providing an optimal balance between sensitivity (76%) and specificity (94%). Moreover, analysis of the remaining AM-RCSQ components reinforces these observations. The ability to return to sleep after an awakening (threshold = 53) demonstrated good diagnostic accuracy (AUC = 0.82; SD = 0.0276; *p* < 0.001). Similarly, perceived overall sleep quality (threshold = 38) yielded particularly convincing results (AUC = 0.83; SD = 0.0215; *p* < 0.001). The Noise item (threshold = 40) showed (AUC = 0.83; SD = 0.0196; *p* < 0.001). However, it is the AM-RCSQ total score (threshold = 49.4) that proved to be the most effective, displaying perfect performance (AUC = 1.00; SD = 0.00; *p* < 0.001) with 100% sensitivity and 100% specificity.

**Figure 1:**
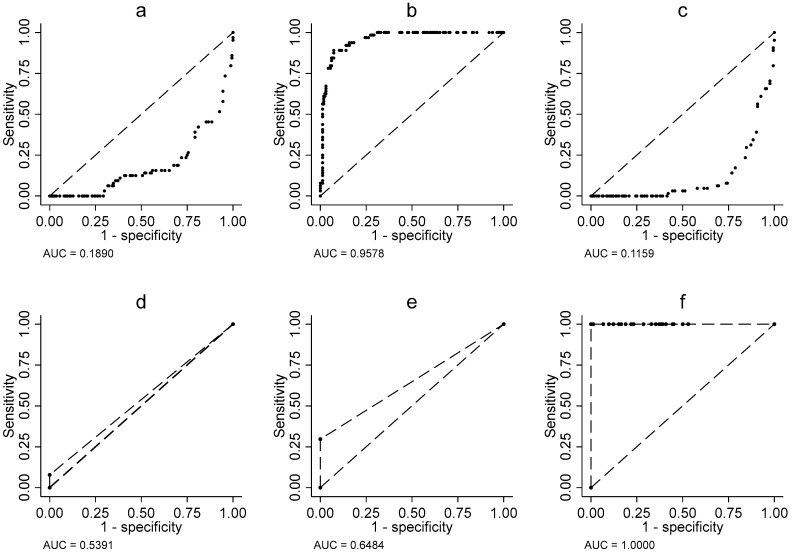
Area under ROC curve, (**a**) Sleep latency, (**b**) Sleep duration, (**c**) Interruption, (**d**) Sleep depth, (**e**) sleep efficiency, (**f**) Score total.

In contrast, actigraphic data presented a more nuanced picture. Although total sleep time (threshold = 277 min) achieved excellent diagnostic performance (AUC = 0.91; SD = 0.0124; *p* < 0.001) (Figure 1b), other parameters such as sleep latency (AUC = 0.50; SD = 0.0314; *p* < 0.001) (Figure 1a) and number of interruptions (AUC = 0.50; SD = 0.0223; *p* < 0.001) (Figure 1c) showed limited discriminative capacity. However, it is noteworthy that the total sleep efficiency score (threshold = 49) matched the exceptional performance of the total RCSQ score (AUC = 1.00; SD = 0.0; *p* < 0.001) (Figure 1f). Conversely, sleep depth measured by actigraphy, with an optimal threshold of 0, exhibited limited discriminative capacity (AUC = 0.54; SD = 0.0169; *p* < 0.020) (Figure 1d), characterized by maximal specificity (100%) but zero sensitivity. Similarly, sleep efficiency, also with a threshold of 0, showed slightly better but still moderate performance (AUC = 0.65; SD = 0.2878; *p* < 0.001) (Figure 1e), once again displaying an imbalanced profile with perfect specificity (100%) but insufficient sensitivity (30%) (Figure 1e). These findings suggest that while sleep regularity may serve as a specific marker, it fails to identify most patients with sleep disturbances.

In summary, these results highlight the overall superiority of the AM-RCSQ as a tool for evaluating sleep quality in hospital settings. They particularly underscore the excellent psychometric reliability of the AM-RCSQ total score, while also acknowledging the specific utility of certain actigraphic parameters, such as total sleep duration. Ultimately, this comparative analysis reveals potential complementarities between the two assessment methods, offering a more comprehensive and accurate evaluation of sleep quality in intensive care patients.

Table 3 compares the demographic, clinical, and sleep parameters between two groups of patients: those with poor sleep quality (*n* = 164) and those with good sleep quality (*n* = 64). This comparative analysis is based on objective actigraphy measurements and clinical data, with means and standard deviations reported for each group, along with *p*-values derived from the Mann–Whitney U test.

The results immediately highlight significant demographic differences. Among participants with poor sleep quality, 57.32% were women and 42.68% were men. In contrast, in the group with good sleep quality, 20.31% were women and 79.69% were men. This difference between groups is statistically significant (*p* < 0.001), indicating a higher proportion of men among good sleepers and a higher proportion of women among poor sleepers. Furthermore, patients in the poor sleep group were older on average (56.09 ± 16.19 years) compared to those in the good sleep group (49.52 ± 17.4 years, *p* = 0.010).

The analysis of clinical scores also reveals substantial differences:

The pain score (VAS) was significantly higher in the poor sleep group (4.79 ± 3.12 vs. 2.22 ± 2.78, *p* < 0.001), as were the Charlson Comorbidity Index (1.45 ± 1.36 vs. 0.77 ± 1.26, *p* < 0.001), the SOFA score (0.82 ± 0.86 vs. 0.5 ± 0.8, *p* = 0.004), and the APACHE II score (11.65 ± 5.65 vs. 8.14 ± 5.26, *p* < 0.001). Regarding sleep parameters derived from actigraphy, the differences between groups were particularly striking: Objectively measured sleep latency was nearly twice as long in poor sleepers (45.2 ± 22.01 min vs. 23.94 ± 13.21 min, *p* < 0.001), total sleep time showed a substantial gap (143.07 ± 103.14 min vs. 339.3 ± 61.34 min, *p* < 0.001), representing nearly 3.5 h of difference, additionally, the number of sleep interruptions was significantly higher in the poor sleep group (60.33 ± 24.35 vs. 31.66 ± 9.48, *p* < 0.001).

## 3. Discussion

Assessing sleep quality in the ICU patients remains a major challenge due to multiple physiological and environmental factors disrupting sleep. Patients in these units are frequently subjected to intensive medical treatments, frequent interventions, and care conditions that impair their ability to achieve restorative sleep [14,15]. This study compared two sleep assessment tools: the Richards–Campbell Sleep Questionnaire (AM-RCSQ), a subjective method, and actigraphy (ACT), an objective method, to determine their reliability and limitations in the ICU setting. The findings revealed poor sleep quality, as assessed by both subjective and objective methods, although the two methods displayed different performances in terms of sensitivity and specificity.

AM-RCSQ results, with an average score of 38.16 ± 17.09, indicate that patients perceive their sleep quality as poor. This score is well below the threshold of 50, which is commonly accepted as the cut-off for good sleep quality. These results are consistent with other studies conducted on ICU patients. Naik et al. reported an average score of 46, suggesting that ICU patients frequently experience sleep disturbances due to the hospital environment and medical treatments, which are often responsible for poor sleep quality [16]. Specific AM-RCSQ parameters such as sleep latency (35.71 ± 21.44 min) and the number of awakenings (40.32 ± 20.03) also demonstrated difficulties in falling and staying asleep findings in line with those of Zang et al. and Ahn et al., who reported elevated sleep latency and increased awakenings in ICU settings [17,18]. However, the use of the RCSQ in ICU settings has important limitations, primarily linked to the clinical condition of patients. Factors such as sedative administration, the presence of delirium, and central nervous system (CNS) dysfunction can impair patients’ subjective perception of their sleep. Talvacchia et al. emphasised that these factors may limit the reliability of patient self-assessments [19]. In this study, AM-RCSQ results highlight clear difficulties in maintaining restorative sleep, directly linked to medical and environmental disruptions.

ACT, used as an objective method to assess sleep quality, showed that the average sleep duration was 198.15 ± 128.42 min, or approximately 3.3 h, well below than the recommended 7 to 9 h for restorative sleep [20]. Similar findings were reported by Nilius et al. and Romagnoli et al., who observed reduced sleep durations in ICU patients, insufficient for recovery [21,22].

Furthermore, the frequency of sleep interruptions was high (52.28 ± 24.85 episodes per night), which also reflects poor sleep quality in this population. Boyko et al. also reported frequent interruptions in ICU environments, which interfere with sleep continuity and disrupt regular sleep cycles [23].

This study also allowed for a comparison between subjective and objective assessments. Both methods indicated poor sleep quality, but the correlation between them was weak. The RCSQ showed area under the ROC curve (AUC) values close to 1.00 for several parameters, such as sleep depth, sleep latency, and number of awakenings, confirming that the RCSQ is a reliable tool for identifying good-quality sleep. In contrast, ACT showed low sensitivity for key parameters, such as sleep latency and sleep interruptions, raising concerns about its effectiveness as a standalone diagnostic tool. These findings are consistent with those of Conley et al., who reported that ACT has lower sensitivity compared to more robust methods, such as polysomnography (PSG), the gold standard for sleep assessment [24].

Regarding BMI, this study observed that patients with a higher BMI had significantly poorer sleep quality. This result aligns with findings from Figorilli et al. and Carneiro et al., who reported a relationship between obesity and sleep disorders. Obesity may contribute to sleep disturbances due to increased body mass and associated respiratory comorbidities, indicating the need to consider BMI as a potential risk factor in ICU sleep assessments [25,26]. As for gender differences, this study revealed that women in the ICU experienced significantly poorer sleep quality than men. Interestingly, Altman et al. reported that men were more likely to experience sleep disorders in ICU settings, possibly due to specific comorbidities or greater susceptibility to treatment effects [27].

Ultimately, while ACT offers benefits as an objective method of sleep assessment, this study confirms that ACT alone is insufficient for evaluating sleep quality in ICU settings. ACT lacks sensitivity and specificity for key sleep parameters, such as sleep latency and sleep interruptions. Future research should aim to improve the accuracy of objective tools, such as polysomnography, while continuing to explore ACT as a complementary method. Thus, combining subjective and objective methods could enable more complete and accurate evaluations of sleep quality in ICU patients. This comprehensive approach may help identify the influencing factors and guide the development of effective strategies to improve sleep in this vulnerable population.

These findings support two key conclusions: first, there is a strong association between sleep quality and various clinical markers; second, sleep disturbances in the ICU are not limited to subjective perception but manifest as objective alterations measurable via actigraphy. Finally, the consistency across demographic, clinical, and sleep-related data suggests that patients with poor sleep quality form a distinct subgroup with specific characteristics, potentially requiring targeted management strategies.

## 4. Materials and Methods

### 4.1. Study Design

The study was cross-sectional and observational. Data were collected prospectively from patients admitted to the ICUs of three hospitals in the Souss-Massa region, located in southern Morocco, during the period from 16 February 2023 to 15 March 2025.

### 4.2. Population and Sampling

Patients were selected using an exhaustive sampling method, including all patients admitted during the study period who met the inclusion criteria. The final sample consisted of 228 critically ill patients admitted to the ICU, aged 18 years or older, who were Arabic-speaking and had provided informed consent. Patients were excluded if they had hearing or speech impairments, a pre-existing diagnosis of dementia (Mini-Mental State Examination-MMSE score < 24), documented substance use disorders, or a Glasgow Coma Scale score below 15.

### 4.3. Data Collection

Sleep characteristics and quality were assessed simultaneously using the AM-RCSQ and ACT. Patient inclusion, actigraphic monitoring, and questionnaire administration were conducted by two nurses with prior training in the use of these instruments.

### 4.4. Nurse Training and Clinical Setting

Data collection was implemented in the Medical ICU at the regional hospital in Agadir. Bedside nurses received standardized training delivered by a sleep specialist consisting of a 120-min didactic module and training. The curriculum covered device initialization, placement of the sensor under the mattress, placement-time guidance and escalation pathways for device issues. Competency was verified using a checklist during the first supervised deployment.

### 4.5. Assessment Tools

#### 4.5.1. AM-RCSQ

Sleep was evaluated using the Moroccan Arabic version of the validated RCSQ (AM-RCSQ). This five-item questionnaire assesses various dimensions of sleep, including perceived sleep depth, sleep latency, number of awakenings, sleep efficiency, and time spent awake. Each item is scored on a visual analogue scale (VAS) ranging from 0 to 100 mm, with higher scores indicating better perceived sleep. The mean of the five items constitutes the global AM-RCSQ score. The psychometric properties of the Moroccan version of the AM-RCSQ have demonstrated high reliability (Cronbach’s alpha between 0.89 and 0.92) and good criterion validity compared to PSG (r = 0.58, *p* < 0.001) [28].

The questionnaire was administered three times during the patient’s ICU stay, always at the same time (9:00 a.m.). On average, the questionnaire took four minutes to complete.

#### 4.5.2. Actigraphy

Sleep was measured with an under-mattress sensor (Withings Sleep Analyzer, Withings, France; firmware 6061). The device combines a pneumatic (ballistocardiography) and an acoustic sensor to capture micro-movements, heart rate, respiratory rate, and snoring; signals were aggregated into 30-s epochs and processed by the embedded algorithm to estimate total sleep time, sleep efficiency, sleep onset latency, wake after sleep onset, and an apnea–hypopnea index.

The device was placed under the mattress for three consecutive nights. Given the study’s focus on nocturnal sleep and its pilot design, data were recorded from 7:00 p.m. to 7:00 a.m.

The associated software, integrated into the Health Mate application, enables the visualization, analysis, and tracking of various sleep parameters, including sleep duration, sleep stages, and nighttime awakenings [29].

The actigraphic parameters analysed are detailed in Table 4.

### 4.6. Sleep Quality

An AM-RCSQ total score ≥ 50 was used to define good sleep (sensitivity 88.24%, specificity 86.67%; receiver operating characteristic [ROC] area = 0.92. Patients with a total AM-RCSQ score below 50 were considered to have poor sleep. This cut-off was established through statistical analysis to determine the optimal balance between sensitivity and specificity. Similarly, a total sleep efficiency score by actigraphy ≥ 50 was used to define good sleep [30].

We specify that AM-RCSQ is the primary criterion for distinguishing between participants with good or poor sleep, while actigraphy metrics, including sleep efficiency, were employed in secondary sensitivity analyses and not as the primary measure.

### 4.7. Ethical Approval

This cross-sectional study was conducted with adult patients and received approval from the Biomedical Research Ethics Committee (CERB) of the Faculty of Medicine and Pharmacy of Rabat (Ref. No. 154/24). Authorisation to collect data was granted by the Regional Department of Health and Social Protection of the Souss-Massa region. Prior to participation, all patients received a detailed explanation of the study and provided written informed consent, in accordance with the ethical standards governing biomedical research [31].

### 4.8. Data Analysis

Descriptive statistics (absolute/relative frequencies, mean, and standard deviation [SD]) were used to analyse demographic and clinical data and to assess the different components of the questionnaire.

Statistical analysis was performed using STATA software version 17.0 (STATA Corporation, College Station Road, Houston, TX, USA). Diagnostic accuracy (binary discrimination ability) of the tools was assessed using ROC curve analysis and Youden’s Index, with a significance threshold set at *p* < 0.05. AUC values between 0.75 and 0.92 indicate good diagnostic capacity, between 0.92 and 0.97 excellent, and between 0.97 and 1.00 excellent diagnostic performance.

The area under the ROC curve AUC for the AM-RCSQ score was not included in Figure 1 because the AM-RCSQ score was primarily used as a categorical variable to differentiate between participants with good or poor sleep, rather than as a continuous measure. In contrast, actigraphy was treated as a continuous variable, which allowed for the calculation of the AUC.

Relationships between certain RCSQ items and actigraphy parameters were assessed using Spearman’s correlation coefficient. Chi^2^ test was used to examine the association between sleep quality and gender. Differences in sleep quality, as measured by the AM-RCSQ and actigraphy, were compared using the non-parametric Mann–Whitney U test, with statistical significance set at *p* < 0.05.

## 5. Conclusions

Accurately measuring sleep quality in ICU patients remains a significant challenge, both in terms of practical implementation and data interpretation. This study demonstrated that the AM-RCSQ, with its excellent discriminative power, is a reliable subjective tool for assessing patients’ perception of sleep quality. Additionally, ACT provided objective data revealing concerning patterns, such as a short average sleep duration, which is well below clinical recommendations. Moreover, significant differences based on gender and age suggest an important influence of demographic factors. However, this research faced several limitations. First, the impact of sedative drugs on actigraphy readings could not be fully controlled. Second, the clinical condition of patients may have influenced their responses to the AM-RCSQ.

Nevertheless, the findings open up important perspectives for sleep management in ICU settings. Future research should focus on two main directions: improving actigraphy algorithms to adapt them to the specific challenges of immobilised ICU patients and developing integrated approaches that combine subjective and objective tools. While the AM-RCSQ has proven effective, its association with other tools appears essential to obtain a comprehensive view of sleep disorders in critical care. This dual approach may lead to more targeted interventions, ultimately improving both the quality of care and patient outcomes.

### Strengths and Limitations

This study presents several important limitations that should be acknowledged. First, the influence of sedative medications on actigraphy data could not be fully controlled, which may have affected the accuracy of objective sleep measurements. Second, the subjective nature of the AM-RCSQ might be influenced by patients’ clinical conditions, such as delirium or neurological impairments, potentially biasing self-reported sleep quality. Third, the study’s setting in a specific region of Morocco may limit the generalizability of findings to other healthcare contexts. Fourth, the cross-sectional and pilot design restricts the assessment of changes over time and causal relationships. Fifth, some potential confounders like concurrent treatments, pain levels, and environmental factors were not exhaustively controlled. Finally, we did not systematically ascertain participants’ preadmission histories of sleep disorders or use of sedative–hypnotic medications. Consequently, we were unable to adjust for baseline sleep phenotypes or medication effects, and residual confounding of both subjective and actigraphic outcomes is possible. Despite these limitations, the study provides valuable insights by simultaneously assessing sleep quality with both subjective (AM-RCSQ) and objective (actigraphy) tools in a relatively large ICU patient cohort. The excellent discriminative performance of the AM-RCSQ total score and the identification of significant demographic and clinical associations highlight the clinical relevance of the findings. Furthermore, this research opens up avenues for improving sleep assessment methods and tailoring interventions to enhance patient outcomes in critical care.

Future studies should build on these results with larger, multicenter, and longitudinal designs to validate and extend the understanding of sleep disturbances in ICU patients, ultimately guiding better management strategies.

## Figures and Tables

**Table 1:** Descriptive statistics of key characteristics and variables of the sample (*n* = 228).

	Variables	Mean ± SD
Patients	Age [Years]	54.25 ± 16.76
	BMI [kg/m^2^]	23.13 ± 4.86
	Pain-score VAS (Visual Analogue Scale)	4.07 ± 3.24
	Charlson score	1.25 ± 1.37
	SOFA score	0.73 ± 0.85
	APACHE II score	10.66 ± 5.76
	Length of Stay [Days]	5.44 ± 2.53
AM-RCSQ	Sleep Depth [Score]	39.03 ± 22.43
	Sleep Latency [Score]	35.71 ± 21.44
	Number of Sleep Interruptions	40.32 ± 20.03
	Return to Sleep After Awakening [Score]	38.88 ± 18.81
	Perceived Sleep Quality [Score]	38.93 ± 17.12
	Noise [Score]	33.23 ± 17.72
	RCSQ Total [Score]	38.16 ± 17.09
Actigraphy	Sleep Onset Latency [min]	39.23 ± 22.09
	Total Sleep Time [min]	198.15 ± 128.42
	Number of Sleep Interruptions	52.28 ± 24.85
	Overall Sleep Quality [score]	37.03 ± 19.2

**Table 2:** ROC Analysis of AM-RCSQ and Actigraphy Parameters for Sleep Discrimination among Participants (*n* = 228).

	Variable	Cut-Off Value ^a^	Youden’s Statistic	AUC (SD) ^b^	*p*-Value ^c^	Sensitivity (%)	Specificity (%)	Accuracy (%)
AM-RCSQ	Sleep Depth	53	0.6711	0.84 (0.0204)	<0.001	82.00	85.00	82.46
	Sleep Latency	43	0.7173	0.86 (0.0130)	<0.001	92.00	80.00	82.02
	Number of Awakenings	57	0.6940	0.85 (0.0257)	<0.001	76.00	94.00	89.04
	Return to Sleep After Awakening	53	0.6341	0.82 (0.0276)	<0.001	73.00	90.00	84.65
	Perceived Sleep Quality	38	0.6575	0.83 (0.0215)	<0.001	96.00	70.00	74.12
	Noise	40	0.6540	0.83 (0.0196)	<0.001	82.00	84.00	73.25
	Total AM-RCSQ Score	49.4	1.0000	1.00 (0.00)	<0.001	100.00	100.00	99.56
Actigraphy	Sleep Latency	97	0.0000	0.5 (0.0314)	<0.001	0.00	100.00	71.49
	Total Sleep Duration	277	0.817	0.91 (0.0124)	<0.001	89.00	93.00	89.47
	Number of Sleep Interruptions	124	0.0000	0.50 (0.0223)	<0.001	0.00	100.00	71.49
	Sleep Depth	0	0.078	0.54 (0.0169)	<0.020	08.00	100.00	0.00
	Sleep Efficiency	0	0.297	0.65 (0.2878)	<0.001	30.00	100.00	0.00
	Total Score (Sleep Quality)	49	1.000	1.00 (0.0)	<0.001	100.00	100.00	99.12

^a^: Cut-off value: Empirical optimal cut point. ^b^: AUC: area under curve at cutpoint, SD: standard deviation. ^c^: *p*-value of Youden’s Index.

**Table 3:** Actigraphy Parameters and Clinical Characteristics by Sleep Quality Groups (*n* = 228).

Variables	Mean ± SD		*p*
	Poor Sleep Quality *n* = 164 (% = 71.9)	Good Sleep Quality *n* = 64 (% = 28.1)	
Gender			<0.001 ^a^
Female (*n* = 107, % = 46.9)	94 (87.85)	13 (12.15)	-
Male (*n* = 121, % = 53.1)	70 (57.85)	51 (42.15)	-
Age [Years]	56.09 ± 16.19	49.52 ± 17.4	0.010 ^b^
BMI [kg/m^2^]	23.23 ± 4.96	22.89 ± 4.63	0.764 ^b^
Pain VAS score	4.79 ± 3.12	2.22 ± 2.78	<0.001 ^b^
Charlson score	1.45 ± 1.36	0.77 ± 1.26	<0.001 ^b^
SOFA score	0.82 ± 0.86	0.5 ± 0.8	0.004 ^b^
APACHE II score	11.65 ± 5.65	8.14 ± 5.26	<0.001 ^b^
Length of Stay [Days]	5.77 ± 2.64	4.59 ± 1.99	<0.001 ^b^
Sleep Onset Latency [min]	45.2 ± 22.01	23.94 ± 13.21	<0.001 ^b^
Total Sleep Time [min]	143.07 ± 103.14	339.3 ± 61.34	<0.001 ^b^
Number of Sleep Interruptions	60.33 ± 24.35	31.66 ± 9.48	<0.001 ^b^
Overall Sleep Quality [score]	28.1 ± 13.91	59.92 ± 9.39	<0.001 ^b^

^a^: Chi^2^ test bilateral statistical significance. ^b^: Mann–Whitney U test bilateral statistical significance.

**Table 4:** Definitions of Actigraphy Parameters.

Actigraphy Parameters	Unit	Definition
Sleep onset latency	Minutes	The elapsed time between wakefulness inhibition and sleep onset
Total sleep time	Minutes	The total time spent sleeping, from sleep onset to final awakening.
Number of awakenings	Episodes	The number of awakenings during the night.
Sleep depth	Score	Duration of the deep sleep phase (NREM Stage 3)
Sleep efficiency	Score	The proportion of time spent sleeping relative to time spent in bed.
Global sleep quality score	Score	Sleep quality, rated on a scale of 0 to 100.

## Data Availability

The datasets generated and analyzed during the current study are available from the corresponding author on reasonable request.
